# Health Promotion for Outpatient Careworkers in Germany

**DOI:** 10.3390/healthcare10061148

**Published:** 2022-06-20

**Authors:** Natascha Mojtahedzadeh, Monika Bernburg, Elisabeth Rohwer, Albert Nienhaus, David A. Groneberg, Volker Harth, Stefanie Mache

**Affiliations:** 1 Institute for Occupational and Maritime Medicine (ZfAM), University Medical Center Hamburg-Eppendorf (UKE), Seewartenstr. 10, 20459 Hamburg, Germany; n.mojtahedzadeh@outlook.de (N.M.); e.rohwer.ext@uke.de (E.R.); harth@uke.de (V.H.); 2 Institute for Occupational Medicine, Social Medicine and Environment Medicine, Goethe University Frankfurt, Theodor-Stern-Kai 7, 60590 Frankfurt, Germany; monika.bernburg@hin.ch (M.B.); groneberg@med.uni-frankfurt.de (D.A.G.); 3 Department of Occupational Medicine, Hazardous Substances and Public Health, Institution for Statutory Accident Insurance and Prevention in the Health and Welfare Services (BGW), Pappelallee 33/35/37, 22089 Hamburg, Germany; a.nienhaus@uke.de; 4 Institute for Health Service Research in Dermatology and Nursing (IVDP), Competence Center for Epidemiology and Health Services Research for Healthcare Professionals (CVcare), University Medical Centre Hamburg-Eppendorf (UKE), Martinistr. 52, 20246 Hamburg, Germany

**Keywords:** health behavior, outpatients, qualitative research, caregivers, Germany

## Abstract

Due to ongoing demographic changes, the need for care is increasing in Germany. The number of outpatient care services is also rising, and with it, the number of employees in outpatient care, who are also continuously becoming older. Workplace health promotion (WHP) becomes relevant in this context, as it can reduce negative strain reactions and promote employees’ health. The aim of this study was (1) to reveal implemented WHP interventions in German outpatient care services; (2) to examine the potential challenges regarding a successful implementation of WHP measures; and (3) to illuminate further requests and needs experienced by outpatient careworkers. In qualitative field research, 30 semi-structured individual interviews were conducted with German caregivers, using the problem-centered interview method. The collected data were deductively and inductively evaluated and interpreted, using qualitative content analysis according to Mayring. Outpatient caregivers reported various WHP measures known from their workplaces, such as the provision of fruit baskets, programmes to increase physical activity, or a subsidy for a personal gym. They further reported WHP, such as back training, known from other care services. However, the respondents spoke of the challenges regarding the implementation or the use of WHP interventions in general. The most frequently named barriers were a lack of time after work and interventions that were only offered in their leisure time. In the same course, the participants still needed offers to increase physical activity, joint activities, or relaxation techniques. However, respondents highlighted that they preferred the interventions to take place during working hours. This way, they would also be more likely to take advantage of the interventions. The results of this study provide an insight into various WHP measures that already exist, or that are desirable for implementation with regard to caregivers’ needs. Subjectively perceived challenges for a successful implementation of WHP measures represent the importance of adjustments in the work organization of caregivers. It becomes clear that WHP is not yet established in the ambulant care sector, although it appears to be imperative for keeping caregivers healthy. Considering the different needs of employees, the results can provide a basis for the development of needs-based health promotion measures for caregivers.

## 1. Introduction

Time and performance pressure, a lack of regeneration phases, and unclear shift scheduling, as well as gratification crises are stress-causing factors in outpatient care [1]. An additional burden is the shortage of skilled workers in the care sector [2]. The risk of interested self-endangerment through overtime and team loyalty may be increased, which can eventually lead to presenteeism [3]. Moreover, the perception of stress is widespread in the care setting [4]. Furthermore, fatigue and stress perception can lead to health limitations and mental illnesses as a result of negative strain reactions [5,6]. Burnout as a long-term consequence of stress is also increasingly common among caregivers [7,8]. This is occurring in the context that people who are in need of care in Germany are increasingly being cared for on an outpatient basis [9], and simultaneously, the sickness rate is increasing among caregivers recorded in Germany [10]. It is important to focus on outpatient caregiver health and health promotion. In addition, among German employees, higher rates of incapacity to work were found among the nursing professions by 2018, compared to other occupational groups [10]. Among employees in the care sector work, disability days were higher in total (22.9 vs. 14.9) [10]. With the increasing relevance of days with incapacity to work due to mental disorders, stress prevention is also of great importance in this context [11,12]. Moreover, outpatient caregivers as an employee group are becoming older in general. The age groups, 30–40 years (94,499, 22.4%), 40–50 years (95,597, 22.7%) and 50–60 years (122,774, 29.1%), represent the highest proportions of employees in outpatient care and care services in Germany [13]. With increasing age, the older population of employees becomes a more vulnerable group [14,15]. In addition to possible occurring comorbidities, older people, and thus, workers, are expected to have worse health behaviors [15,16].

Looking at the care sector and its known job demands, work health promotion (WHP) becomes relevant in the care setting. WHP can, for example, be aimed at strengthening personal, social, and organizational resources: activities that are tailored to personal skills and social exchange, as well as the creation of possible scopes for decision-making and action. Training at the behavioral level, e.g., learning how to protect the back when moving and transporting patients, as well as the creation and facilitation of regeneration and retreat opportunities, can contribute to health-promoting behavior. Health counselling (e.g., in relation to nutrition) can also be effective in terms of improving healthy behaviors [17]. However, as biopsychosocial beings, people are also susceptible to (adverse) working conditions [18]. For this reason, a combination of behavioral and structural-oriented prevention measures, as well as target group-specific orientations must be a premise. Thus, working conditions in the care and the health status of all employees must be taken into account in the development and implementation of interventions to maintain and to improve health [19].

### 1.1. Current State of Research

There are already intervention studies examining the effects of behavioral WHP measures in the care setting (e.g., [20,21,22,23,24,25,26,27,28,29,30,31,32,33]), mostly focusing on stationary nursing staff, except for Glashütter [30] (outpatient caregivers from Austria) or Craigie, Slayter, Hegney, Osseiran-Moisson, Gentry, Davis, Dolan and Rees [24] (also including outpatient care). Recently published results from Germany identified, among other things, WHP offers in the German care setting from the perspective of experts (e.g., management and patient caregivers, etc.). Individual counselling services, such as smoking seminars, nutrition counselling, programmes for stress management, and resilience enhancement, as well as fruit baskets, were reported by the experts [34].

Muramatsu, Yin and Lin [20] implemented a physical activity session for home care patients in their pilot programme, with the aim of increasing the health skills and positive health behaviors of the workers. For four months, the nurses reminded the patients of the exercise sessions that they had learned (exercises for the back, arms, and legs). The intervention was able to contribute to self-motivation and increase the nurses’ personal physical activity after the intervention, by applying what they had learned themselves, and by increasing their knowledge regarding physical activity. Moazzami, Dehdari, Taghdisi and Soltanian [31] used a randomized control trial (RCT) to test an educational intervention focusing on ergonomics to observe how it affected nurses’ bodily behavior. The intervention group showed a better body posture at work, and thus reduced demands [31]. Flanagan, McCord, Cheney and Lundquist [32] provided a wireless pedometer to nurses to test whether the daily step count changed. A significant increase (*p* < 0.001) in physical activity was observed [32]. In an Austrian WHP project involving ambulatory care workers, body awareness seminars, and back training helped to reduce physical complaints by 12%, and specific shoulder complaints by 13%. Overall, 37% of the participants improved their physical activity behavior. In addition, 44% of the participants positively changed their eating behaviors as a result of a nutrition programme (seminars on healthy eating and organized community runs) according to self-reporting [30]. Stress reduction by increasing mindfulness is a frequent target of behavioral interventions. In inpatient care, a 7-week intervention (one session per week) was conducted during working time in a randomized controlled trial involving German nurses aged >45 years, which led to a significant improvement in mental health in the intervention group Maatouk, Mueller, Schmook et al. [21]. Mahon, Mee, Brett and Dowling [23] evaluated a mindfulness intervention (6- to 8-week training) in their pilot study. There was a significant reduction in stress among nurses. In addition, a strengthening of compassion was indicated. Another intervention introduced a workshop day on compassion, and further weekly training focused on mindfulness and resilience promotion. There was a significant reduction in stress perception and burnout risk among outpatient and inpatient nursing staff [24]. The widely used Mindfulness-Based Stress Reduction (MBSR) programme from Jon Kabat-Zinn, which aims at individual stress management by increasing mindfulness, brought about an improvement to the general state of health of nurses in partial telephone sessions [25,35]. An implementation of the MBSR programme in psychiatry showed a significant reduction in work stress perception, and a reduction in the risk of developing depression and anxiety disorders among nurses [26]. The risk of burnout could be reduced through mindfulness, meditation, and yoga interventions for doctors and nurses [27]. Another group intervention (1 h/week, 8 weeks) also including music besides yoga movements and meditation, decreased stress among intensive care nurses [33]. Interventions to increase caregivers’ self-compassion can contribute to reductions in stress perception and resilience promotion [24,28]. A smoke cessation intervention offered 10 sessions (two times/week, three follow-ups) in five groups. The focus was on education regarding addictive behavior and possible obstacle factors, increasing self-efficacy, and stress management and relaxation techniques. After the last session, almost 50% of the participants stopped smoking, and 25% of the respondents reduced their number of cigarettes per day [29]. Power et al. [36] emphasize the needs analysis in this context. The WHP intervention, which was developed on the basis of the personal opinions of nurses, was target group-oriented and needs-based, and showed an improvement in physical activity and eating behaviors among the participants [36].

As the creation of healthy working conditions by conducting WHP measures targeting the structural level is also relevant [37], this has already been aimed at in several studies (e.g., [34,38,39,40,41]). In the care sector, a health-promoting approach on the structural level was implemented; for example, through provision and instruction on the use of a massage chair in the common room. Over a period of 6 months, the stress experiences of nurses was significantly reduced [38]. With regard to the shortage of skilled nursing staff, an improvement of working conditions, as well as an increased deployment of staff in inpatient nursing care has already proven to improve stress management and nursing performance [39]. A perspective on the increased loss of breaks in care (cf. [42]) and adapted working conditions can have a favorable effect on taking breaks. For instance, Nejati, Shepley, Rodiek, Lee and Varni [40] emphasized this circumstance by highlighting the close location and design of a break room in inpatient care. Meal breaks were mostly spent in the rooms provided. This significantly reduced the perceived stress levels of the nurses [41]. The experts interviewed from the German care setting also considered the rostering of care workers as a WHP measure at the organizational level [34].

Riedel-Heller et al. [43] emphasize that a combination of measures at the behavioral and structural level should be aimed at in order to effectively reduce undesirable burdens in the workplace. The “Be Well, Work Well” intervention, developed at the Harvard T.H. Chan School of Public Health, Center for Work, Health and Wellbeing—showed unexpected results: despite implementing measures at the ergonomic, organizational (e.g., optimized break organization) and personal levels (e.g., the promotion of health-promoting behavior), the intervention was not been successful for decreasing the perceived stress levels of inpatient nursing staff. The main reason for this was a lack of time [44]. Sports, nutrition, and stress management programmes were offered in Taiwan in one hospital; however, inpatient nurses made less use of them compared to other employee groups [45]. The “COMPASS” programme (“Community of Practice and Safety Support”) by Olsen et al. [46] is specifically designed for outpatient caregivers in America. It is designed to increase a sense of community among each other by setting weekly group goals related to nutrition (healthy recipes). It also consists of teaching units, such as education on health promotion, occupational health and healthy eating, and fitness programmes. The pre/post comparison showed significant improvements in health status after 6 months in health status, and a reduction in the stress levels of the participants [46]. WHP measures that went beyond the optimization of nutrition, exercise, and smoking behavior to work organization and psychosocial stress (e.g., through wellness programmes) were very well received by nursing staff from various old people’s homes, with preference being given to measures offered during the working hours [47].

In summary, there are neither studies that only focus on existing WHP measures in outpatient care in Germany, nor research regarding outpatient caregivers’ wishes and needs in terms of WHP, except for Ehegartner et al. [48], who also partially included inpatient and outpatient caregivers, and Neumann, Mojtahedzadeh, Harth, Mache, Augustin and Zyriax [34] who elicited WHP offers only from care experts’ point of view. In addition, there are no studies including the health impairment of outpatient caregivers in the context of WHP.

As a consequence of the different workplace settings and the associated framework conditions that could influence inpatient and outpatient caregivers’ health behaviors, study results from inpatient care are only generalizable to a limited extent. Ultimately, there are no studies analyzing WHP specifically for German outpatient caregivers, or that focus on the factors that might have an inhibiting or promoting effect in this context. Nevertheless, in terms of the German Occupational Health and Safety Act (ArbSchG) [49]), employers are responsible for ensuring the health of their employees; hence, it is of great relevance to identify existing WHP measures as well as the corresponding needs of outpatient caregivers for developing further WHP interventions, as the need for action regarding WHP in the care setting is evident (cf. [50]).

### 1.2. Study Aims and Research Questions

The aim of this study was to highlight the implemented WHP interventions in German outpatient care services, to examine possible challenges for a successful implementation of WHP measures, and to shed light on further requests and needs experienced by outpatient caregivers.

We proposed the following research questions:

1. How is individual health impairment subjectively perceived by outpatient caregivers?
2. Which specific workplace health promotion measures are already implemented in outpatient care services?
3. Which challenges occur for a successful implementation of WHP in outpatient care services?
4. Which requests and needs are experienced by outpatient caregivers regarding WHP?

## 2. Materials and Methods

### 2.1. Study Design

The present study followed a qualitative research approach by using the problem-centered interview method (PCI), in order to obtain the initial findings of an as-yet unknown field of research [51]. Prior literature research, as well as 6 participatory observations of different working shifts of outpatient caregivers, were conducted to gain knowledge in developing the questions being asked in the interviews [52]. Interviews were conducted with a combination of face-to-face interviews (*n* = 13) and telephone interviews (*n* = 17).

### 2.2. Participant Selection and Interview Conduct

Participants were recruited from 48 different outpatient care services in Northern Germany. The outpatient care services differed in size and the number of employees. In total, 30 semi-structured interviews with outpatient caregivers were conducted using a deductive–inductive procedure [53,54]. Due to the accessibility issues of outpatient caregivers, 17 of the interviews needed to be conducted via telephone. Study participation was voluntary for the outpatient caregivers. Each participant was asked to sign a declaration of informed consent regarding performance and recording, prior to the interviews. All interviewees were in a position to understand and to consent to the study requirements, and they were provided written informed consent. A sampling procedure was applied purposefully. Outpatient caregivers who were working in outpatient care for at least six months in the same outpatient care service (in small and medium-sized enterprises in Hamburg, Germany), and who were fluent in the German language, were eligible and were recruited. Outpatient care services were contacted via invitation emails and telephone calls. Afterwards, all interviews were tape recorded. The interview length ranged from 21 up to approximately 70 min. Participants had the opportunity to terminate the interview at any time. No non-participants were present during the interviews. No repeat interviews were conducted. Field notes were made immediately after each interview.

### 2.3. Interview Guidelines

A semi-structured interview guide was designed within the general framework of the empirical and theoretical background. Questions for the interviews were collected, reviewed, and sorted, and afterwards, they were subsumed into categories [55,56]. In accordance to Misoch [57], the interview guideline was divided into four phases (information phase, warm-up phase, main phase, and end of interview). Table 1 depicts an extract of the interview guidelines. To receive feedback from research colleagues, and to improve the interview guideline where applicable, a pre-test interview was performed before the actual first interview.

### 2.4. Data Analysis

Following Kuckartz [52], all audio recordings of the interviews were transcribed verbatim. The data analysis was conducted in a deductive–inductive process, according to Mayring’s qualitative content analysis. Since communication with the interviewees should be analyzed in a systematic, rule-guided, and theory-based way [58], according to the qualitative content analysis of Mayring [59], all transcripts were anonymized and analyzed in a deductive–inductive process, i.e., the main categories were retrieved deductively on the basis of the interview guideline. Moreover, sub-categories were developed inductively in an iterative process on the basis of the material [60]. MAXQDA 2020 (VERBI Software, 2019, VERBI GmbH, Berlin, Germany) was used for the data analysis [61]. The researcher identified and refined the codes, categories, and sub-categories in an iterative process. Coding was reviewed reciprocally for accuracy, and was purposefully debated with the head of the research group until consensus in terms of the final coding system was reached. The final coding system was displayed in a separate document. The material was then further decreased and compressed. While analyzing, reflexivity and transparency were maintained in relation to the potential influence of the researchers’ goals and biases on the findings, as well as on the interpretations.

## 3. Results

### 3.1. Sample Characteristics

Respondents were between 20 and over 61 years old, as depicted in Table 2. Of 30 interviewed outpatient caregivers, 9 were male and 21 worked full-time. At the time of the survey, the work experience of the interviewees varied, with a range from 1 up to over 10 years. Most of the 30 interviewees were qualified as geriatric nurses or caregivers. Interviewees #10 and #12 had other qualifications that were not relevant to their profession (storekeeper and interior decorator). According to their answers, they slipped into the care profession while working as temps.

### 3.2. Health Impairment of Outpatient Caregivers

Some of the interviewees reported health problems such as slipped discs, rheumatism, or cervical spine problems.

“*Yes, I have a very slight slipped disc. But I don’t know if it’s from nursing, because I’ve always dealt with it professionally*”.

*(#9, outpatient caregiver, ≥61 years, >10 years of outpatient care experience)*

The outpatient caregivers experienced the influence of their work in different ways. Most of them found their job stressful and described an increased experience of stress. Many of them also considered their back problems to be the result of heavy lifting and carrying during their job. Sitting in cars and riding bicycles were also often perceived as being detrimental to health.

“*And, yes, when there’s a traffic jam or I’m sitting in the car for too long, of course I get a backache*”.

*(#15, outpatient caregiver, 20–30 years, 1–5 years of outpatient care experience)*

A few of the outpatient caregivers interviewed also reported showing up for work when they should have called in sick. According to the interviewees, the reasons for this were, for example, that a cold was not considered serious enough to call in sick. They also did not want to leave their waiting patients without help. In this context, many of the interviewees reported having a guilty conscience towards colleagues, or even feared negative reactions from the rest of the team. Existing understaffing or other sick leave in the company also favored this presenteeism behavior.

“*Yes, exactly, simply because it/ because I found it totally awful to have to call in sick somehow and because I also know exactly what the reactions are. They are not/they are mostly, which is a real pity, often not in such a way that you understand, but that you just somehow get a snub or whatever. That’s kind of the way it is, yes. So both things, right? So that and also that you know: ‘oh God, the poor colleagues have to pay for it now’ and those are the things where you think: ‘can’t you just do it?*”

*(#20, outpatient caregiver, 41–50 years, 6–10 years of outpatient care experience)*

### 3.3. Workplace Health Promotion Measures

From the interviews, four main categories relating to WHP were identified: Existing health promotion/occupational health and safety measures by the employer, the knowledge of other offers regarding workplace health promotion, and the use of digital offers and challenges in practice.

#### 3.3.1. Existing Workplace Health Promotion Measures in Outpatient Care Services

There was a variety of offers for workplace health promotion. Most of the interviewed outpatient caregivers reported that their employers provided a fruit basket or beverages for free use on a regular basis. Furthermore, some respondents mentioned internal health promotion offers for smoking or alcohol cessation. Offers to promote physical activity (in-house sports sessions with equipment provided) or mindfulness (e.g., in the form of yoga) were positively highlighted by the outpatient caregivers.

“*Our employer, he provides us with drinks, coffee, he makes a fruit basket every week, so from that point of view we actually live in luxury here*”.

*(#11, outpatient caregiver 51–60 years, 6–10 outpatient care experience)*

One interviewee reported on the company’s cooperation with a nutritionist. This expert could be contacted if necessary, and individual advice could be obtained. Many outpatient caregivers emphasized that they had the opportunity to make suggestions to their employer regarding workplace health promotion measures that could potentially be taken into account. A financial contribution to the gym by the employer was also described as a workplace health promotion measure by the respondents.

“*There are nutritionists we work with. So I could call a nutritionist, I would say ‘I have this and this problem, do you have a tip for it?’ (…) we always have fresh fruit here, smoking cessation, other things, we also had alcohol cessation once. We had several colleagues who were alcoholics and they stopped at some point*”.

*(#23, outpatient caregiver, ≥61 years, >10 years of outpatient care experience)*

The outpatient caregivers interviewed described the occupational health and safety that had been provided by the company in the form of personal protective equipment (PPE) as a perceived WHP measure. In addition, some respondents reported regarding a company doctor that they could consult if necessary.

“*So the company doctor, she’s really easy to contact, even if there’s something wrong, she’s always available/always has an open ear*”.

*(#5, outpatient caregiver, 51–60 years, >10 years outpatient care experience)*

Finally, however, a few outpatient caregivers also mentioned that measures for workplace health promotion or occupational safety were completely absent in their company.

“*No, none at all*”.

*(#8, outpatient caregiver, 20–30 years, 1–5 years outpatient care experience)*

#### 3.3.2. Knowledge of Further Offers Regarding Workplace Health Promotion

Some of the outpatient caregivers interviewed were aware of other workplace health promotion measures, independent of their care service. In this context, the majority of the interviewees reported regarding a back college in which back-friendly work was learned or deepened. Physiotherapy and riding bikes offered during work shifts were also mentioned. Many other outpatient caregivers mentioned that they knew about massage chairs in other care services. Other interviewees mentioned a water dispenser in the company, nutritional counselling, or cooking together.

“*I know that there are workplaces that cook communally, it’s even compulsory once a month, I think it’s like that. What else? I know companies that only have bicycles, that say right from the start that we don’t have cars here, which I think is very good. Unfortunately, it can’t be avoided here. There are companies that offer/Yes, exactly. One company even has massage chairs that are not used. Yes. I know that for example, right?*”

*(#7, outpatient caregiver, 51–60 years, >10 years outpatient care experience)*

#### 3.3.3. Use of Digital Offers in Terms of Workplace Health Promotion

With regard to the use of workplace health promotion measures in digital form, the preferences varied. Some outpatient caregivers reported digital WHP measures that they used on a daily basis, such as pedometers or online programmes for muscle relaxation, back motivation, and exercise stimulation. The majority of respondents, however, expressed explicit disinterest. This was due to a general rejection of apps. Many emphasized that these were more suitable for the younger generation with regard to WHP measures.

“*I generally believe that this is the future, that’s just the way it is. Especially when I see the pupils, the younger ones who are coming up, I think that they are more into it. Everything that I can take with me on my mobile phone is wonderful, I have it with me. I think that has a future. But as I said, it annoys me more. Me, I can’t be impressed with it, I’d rather talk face to face than via an app like that. But I wouldn’t rule it out, (…). Well, I do think that, that has an influence and can have an influence, yes*”.

*(#18, outpatient caregiver, 41–50 years, 6–10 outpatient care experience)*

Table 3 sums up all of the WHP measures mentioned by the interviewed outpatient caregivers.

#### 3.3.4. Challenges in Practice for the Implementation of Workplace Health Promotion Measures

The interviewed outpatient caregivers highlighted some challenges regarding implementations in the practice of WHP measures. Most of them mentioned that they had little free time due to long working hours and shift work in this context. When using potential WHP measures, individual free time should not be affected. Thus, most respondents expressed their preference for WHP measures to be offered during working hours, rather than after work or on their free weekends. Furthermore, disagreement regarding the type of WHP was mentioned among colleagues in the team. Some interviewees also described the partial lack of support on the part of employers with regard to financial components, or the lack of competence on the part of superiors as being difficult.

“*Yes, well, if it was on my days off, of course not. I’m not going there again just to take part in it*”.

*(#27, outpatient caregiver, 41–50 years, 1–5 years outpatient care experience)*

### 3.4. Requests and Needs Regarding Workplace Health Promotion Interventions

The wishes and needs of the interviewed outpatient caregivers were specifically reflected in WHP measures. Many caregivers expressed their wish for sports offers, or subsidies for individual fitness studios. Furthermore, many of the outpatient caregivers interviewed wanted regular low-threshold nutritional counselling, as well as the supply or provision of water by their care service. In addition, the need among the respondents for joint activities to strengthen team spirit was high and often desired. Less frequently, but nevertheless, special relaxation techniques (such as yoga or tai chi) and massages were requested. With regard to occupational health and safety by the care service, it became clear that in the future, outpatient caregivers wanted the employer to provide them with work clothes that they had been lacking up to now.

“*Yes, that maybe you do it together in a group maybe once a week or sit down together or cook together or something*”.

*(#2, outpatient caregiver, 41–50 years, 1–5 outpatient care experience)*

Table 4 provides an overview of all the requests and needs named by interviewed outpatient caregivers regarding WHP.

## 4. Discussion

### 4.1. Discussion of the Interview Results

By conducting our qualitative interview study, we were able to gain insights into the implemented WHP measures in the present outpatient care services in Germany, as well as into the deficiencies and the perceived needs regarding the WHP interventions of the surveyed outpatient caregivers. In addition, challenges for implementation in practice could be illuminated, for instance, regarding the offered time slots. The interviewed outpatient caregivers reported different implementations of WHP measures offered by their employers (e.g., fruit baskets as a free provision of fruit to promote healthy eating during breaks) or they were told about different WHP interventions that they had further knowledge of (e.g., physiotherapy). Moreover, some of the interviewed outpatient caregivers reported on health impairments, such as existing back problems.

#### 4.1.1. Health Impairment, Stress Perception, and Presenteeism

Some of our interviewees reported musculoskeletal problems, which appears to be very common in the German care sector. The number of days of incapacity to work among German caregivers is constantly rising, due to musculoskeletal complaints [12]. Moreover, our respondents perceived their work as being partially stressful. In this context, there also seems to be a relation between the perception of stress and musculoskeletal complaints, as they may facilitate each other [62,63,64]. Furthermore, many respondents were informed about going to work when they actually needed to call in sick. Research findings from German elderly home services showed that in addition to musculoskeletal complaints, the occurrence of presenteeism in elderly care was very widespread. The reasons for this were a particularly high work density and low work-related control, as well as low reward [65]. However, there seems to be a potential for presenteeism that is reduced by the implementation of WHP, which increases the relevance of its development [65,66,67]. Nonetheless, presenteeism in the German care setting has so far been rarely studied. Thesis research results from a study on geriatric nurses from Dresden, Germany, suggest that high work demands and possible negative strain reactions, such as stress perception from the job, could promote presenteeism [68].

#### 4.1.2. Specific Existing Workplace Health Promotion Measures

The majority of outpatient caregivers interviewed reported the free provision of fruit (“fruit baskets”) at work in the context of WHP by their employer. However, it must be mentioned at this point that it takes an average of 66 days for individuals to adopt and to implement healthy behaviors, such as healthy eating, in the long term [69]. Study results from inpatient care underline that working conditions in the care setting (e.g., long working hours) can make the implementation of healthier diets more difficult [70], or that it takes several months before improvements become noticeable [71]. Apart from the fact that an existing fruit basket is no guarantee for increased job satisfaction and thus, productivity [72], its voluntary nature can lead to offers not being made use of [73]. However, WHP interventions from the inpatient setting show that the intake of fruit and vegetables can increase among caregivers, provided that regular training is targeted and supervised [74]. Additionally, according to self-reporting by outpatient caregivers from Austria, their own dietary behavior changed positively as a result of a regular intervention unit [30]. One interviewed outpatient caregiver mentioned the existence of in-house cooperation with a nutritionist. The consultation of nutrition experts also showed success among inpatient nurses when used over a long period of time [75]. The COMPASS project also showed an improvement in health status in outpatient care in the USA, through lessons on healthy eating [46]. In addition, smoking cessation services were mentioned by our respondents, which have also been applied in in-patient hospital settings, and have shown improvements in health-damaging behaviors among nurses and healthcare workers [29,76,77]. Moreover, our interviewed outpatient caregivers mentioned WHP interventions regarding an increase in physical activity, e.g., through on-site offers in the company, or through contribution subsidies for a fitness studio. In this context, many successful interventions have already been used and implemented in the care setting, all with the aim of increasing the physical activity of nursing employees in the long term [20,32,70,71,74,75,78,79,80,81,82,83]. In several studies on inpatient care, the provision of a pedometer among nurses led to more steps per day, and thus, increased general physical activity [32,80,83]. The study results of Lavoie-Tremblay, Sounan, Trudel, Lavigne, Martin and Lowensteyn [80] even showed a reduction in the perception of stress in this course. Likewise, the physical activity intervention of Freitas et al. [84] among nursing professionals, with reductions in depressive symptoms and burnout, were also observed. Our respondents highlighted mindfulness programmes and yoga courses. The use of such interventions in the inpatient care sector, and their success in reducing stress perception, are underlined by other research findings among nurses [21,23,25,26,33,85,86,87,88]. Some interviewed outpatient caregivers spoke about a company doctor who could be contacted if necessary, in this context. In German companies, there is a duty to cooperate with company doctors and occupational safety specialists. These are there to provide support within the framework of occupational safety and accident prevention [89].

#### 4.1.3. Knowledge Regarding Further Workplace Health Promotion Interventions

Most of our respondents reported their knowledge regarding back college, focusing on back-friendly work, as offered by other employers. Previous research results from inpatient nursing settings focusing on back pain reduction show positive effects in nurses through regular intervention sessions, such as education or ergonomic workplace design [31,44,90,91]. Results from Germany are available from the geriatric care sector of Kozak et al. [92]. The implementation of a programme for a reduction in musculoskeletal complaints, with a focus on education and a change of stressful postures, was investigated. After an implementation period of 6 months, it was able to contribute to a reduction in negative postures during work, and thus, to an improvement of the subjectively perceived state of health [92]. The effectiveness of back training was also shown in large evaluation studies among nursing staff [93,94]. Kusma, Pietsch, Riepenhof, Haß, Kuhn, Fischer and Nienhaus [93] recently examined the effectiveness of back training among N = 570 nurses (47.5% trained nurses, 17.7% geriatric nurses, and 11% nursing assistants) from Germany. After 6 months, participants reported fewer musculoskeletal complaints due to the regular implementation of back-friendly work practices from back training in their working and everyday lives. In addition, the reported number of sick days generally decreased, and an improved subjectively perceived state of health were also stated by the nurses [93]. Some of the interviewed outpatient caregivers spoke about a massage chair in the context of further known WHP interventions. This is also known from the care sector, and also showed a stress-reducing effect on nurses [38,95,96,97]. While a massage chair, as reported by other WHP measure participants, might be difficult to arrange in a closely timed and mobile work setting, a water dispenser may be more easily set up. Nutrition counselling could even be combined with cooking with colleagues. During the ongoing pandemic, these offers could even take place digitally.

#### 4.1.4. Use of Digital Workplace Health Promotion Measures

Fewer respondents commented on digital WHP interventions. On digital platforms, online programmes regarding muscle relaxation and sports exercises were more likely to be used. In contrast, however, further scientific evidence shows that digital WHP programmes in hospital settings could contribute to an improvement in mental health, and a reduction in perceived stress [98,99,100,101]. Internet-based WHP also contributed to health-promoting health behaviors among nurses [102]. Finally, it must be emphasized at this point that the outpatient caregivers that we interviewed did not report WHP interventions (neither digital, nor analogue) that focused on stress reduction. This seems unusual, as the reduction in stress perception as an intervention goal, e.g., by increasing mindfulness, is already widespread from the inpatient care setting (e.g., [21,23,24,25,26,28,38,99,103,104,105,106,107,108]). Overall, WHP interventions aiming at eating behavior, physical activity, or stress have a tendency of showing positive outcomes regarding nurses’ health in general [109].

#### 4.1.5. Challenges in Practice for the Long-Term Implementation of Workplace Health Promotion Measures in Outpatient Care Services

As outpatient care has specific characteristics regarding working conditions, e.g., a constant change of setting, staying with patients, or in the transportation vehicle, it can be assumed that the implementation of WHP interventions is generally difficult [17,50,110]. The most frequently mentioned barrier to participating in offered WHP interventions by our interviewed outpatient caregivers was the time at which it (would have) taken place. This is consistent with research findings from the German care setting [48], or from results from the abovementioned “Be Well, Work Well” intervention by Sorensen et al. [44]. Ilvig et al. [111] examined female healthcare workers, who eventually also named time as being the most frequent barrier for attendance. Most of our interviewees were female, so family commitments outside working hours cannot be ruled out, which could prevent the use of WHP programmes outside of working hours as well [48]. A general lack of time, e.g., due to shift work, could be a reason for not using interventions (cf. [44]), as was also mentioned by our respondents. Our interviewed outpatient caregivers also highlighted that a lack of agreement with colleagues on the type of WHP interventions could make them difficult to offer. Low social and financial support, especially on a management level, was also described as being challenging. These barriers are also reflected in research findings from nursing homes [47]. A quantitative study by Otto et al. [112] analyzed different care settings in Germany (elderly care, home care, hospitals, and trainees, N = 242) in terms of work-related burdens, and therefore, the requirements for WHP measures. As nurses differed in their perceived stress levels and well-being, their study highlights the creation of WHP interventions, which are specifically geared toward individual target groups [112]. However, a qualitative interview study with seven managers from outpatient care services in Germany shows that WHP measures are accepted by employees, but there is no structured health management strategy in place [113], which makes the implementation of WHP interventions challenging [114]. Moreover, staff shortages and a lack of skills could also hamper the implementation of WHP interventions [50].

#### 4.1.6. Requests and Needs Regarding Workplace Health Promotion Interventions

The outpatient caregivers interviewed were particularly in favor of WHP programmes to increase physical activity, joint activities, and relaxation techniques, as well as massages. Intervention studies focusing on increasing the physical activity of nursing staff do exist in the scientific literature (cf. [109]). However, their wishes and needs do not fully coincide with other published studies. For instance, the results of a questionnaire study from Germany by Ehegartner, Kirschneck, Frisch, Schuh and Kus [48] targeting nursing staff in outpatient, partly inpatient, and inpatient facilities and clinics (N = 1381), however, revealed that educational training regarding stress (>80%), communication, and team work (>70%) are wished for by caregivers. In addition, approximately 70% wished for passive regeneration units or practical WHP measures such as back training (approximately 60%) [48]. Further study results from inpatient care settings reveal that WHP interventions targeting nurses’ mental health are particularly needed, closely followed by nutritional interventions or interventions to increase physical activity. While smoking cessation was mentioned least frequently, stress reduction and resilience promotion were highlighted most frequently [115]. Faller and Reinboth [116] further underline the risk of burnout in inpatient geriatric care. A needs-based and targeted WHP is indispensable [116]. Nonetheless, an existing need for action on WHP in care remains an issue of no dispute according to Krupp, Hielscher and Kirchen-Peters [50]. A team-building intervention on inpatient nurses also showed in a pre/post comparison that support from supervisors can promote work engagement [117]. A higher degree of work engagement by providing supervisor support is also shown among Malaysian nurses [118].

### 4.2. Strengths and Limitations

A strength of our study is that we have been able to recruit a solid number of outpatient caregivers from different care services of different city districts from Northern Germany. They also had different socio-demographic characteristics, e.g., different ages and lengths of work experience, as well as types of employment. Moreover, recruitment was conducted over a short time period. Ultimately, we were able to establish a broad picture of existing WHP measures of German outpatient care services, as well as outpatient caregivers’ further individual needs and wishes regarding WHP, which has not yet been examined. Giving rich descriptions of our results and displaying many direct quotes from the interviewees increased the trustworthiness of our findings [119]. Additionally, the research findings were discussed in depth in a research group, and contrasted with empirical evidence. Overall, we were able to gain initial findings from this currently unexplored topic. Moreover, we were able to successfully close the research gap identified above.

As limiting factors of our study, it should be mentioned that our study comprised a relatively small sample size; therefore, the results need to be reconsidered in terms of transferability and generalizability [53,120]. However, individual statements of interviews can be significant, and data saturation seemed to have been achieved, since this usually occurs within the first 20 interviews [54,120]. Nevertheless, external validity (transferability) of the study is not given because the sample was too small and thus, it was not representative and the results cannot be transferred. The results of this qualitative study should be verified by further studies with larger sample sizes, particularly via quantitative studies. In this way, broader knowledge on this topic could be provided. The chosen random sample and risk of self-selection through the snowballing technique might be increased, as more interest in the topic can raise participation in studies. Additionally, female nurses tend to be more likely to participate in studies than male nurses. This is also reflected in our study, although it must be mentioned that more women work in German care services than do men [121,122,123].

Further methodological limitations could be seen in the combination of face-to-face interviews and one-to-one telephone interviews. A lack of eye contact, a distanced conversation atmosphere, and a decrease in possible social clues, as well as an asynchronous communication could be the result of interviews that are conducted over the telephone [124,125,126].

### 4.3. Implications for Further Research and Practice

#### 4.3.1. Recommendations for Future Research

Further research studies with larger sample sizes are needed, as outpatient caregivers depict a special group of employees who are facing several demands in their work life, such as having to fill in for sick colleagues, a lack of support and communication, a high workload and time pressure, and therefore, a higher level of risk for negative strain reactions, such as stress [4,127,128,129,130,131,132]. Therefore a target group-specific WHP that is aimed at counteracting possible negative strain reactions caused by workplace-related demands is of particular relevance here (cf. [116]). In such studies, extended questions should be inquired about within the general context of WHP. In addition, barriers to sustainable implementation should be researched, in order for them to be counteracted for long-term health promotion, and to ensure the health of employees in outpatient care. Moreover, this could be a future interest in research, not only expand the sample size, but also to achieve a more representative study sample (e.g., characteristics that should be considered could be different ages and an even gender distribution), using a quantitative questionnaire study. Eventually, it would be of scientific interest to enlighten the perspective of outpatient caregivers’ perceived work-related demands and resources (cf. [133]), and their views regarding potential barriers and support possibilities for a successful long-term implementation of WHP interventions. Finally, it would be interesting to conduct interviews with outpatient caregivers who have experienced the COVID-19 pandemic, to investigate how their employers acted, and to what extent the situation of WHP might have changed as a result of the pandemic; similar to Hetzmann et al. [134], who focused on occupational health and safety measures in German outpatient care services. Specific health promotion/occupational health and safety measures are necessary, especially in the time of the current COVID-19 pandemic, as proposed by Hetzmann, Mojtahedzadeh, Nienhaus, Harth and Mache [134]. After additional research has been conducted, specific interventions within the framework of work-side health promotion and occupational health and safety could be developed and implemented, as specific job demands could be addressed. In this framework, the perceived challenges and influencing factors regarding the long-term use of WHP measures could come to light, which received less attention in the present interview study. Based on this, WHP interventions could be designed and evaluated in such a way that they are used over a long-term and large scale, in order to maintain or promote the health of outpatient caregivers during the course of their work.

#### 4.3.2. Practical Implications

As with the WHP interventions, implications for further practice can divided in two sections, both at a behavioral level and at a structural level [43,135,136].

Outpatient caregivers should be educated regarding healthy behaviors (cf. [43]), as a higher degree of health literacy could imply more health-promoting behavioral patterns by implementing knowledge in practice [137]. In this context, outpatient caregivers could be, for instance, educated regarding healthy diets [138], the potential risks of tobacco consumption (e.g., an increased risk of cancer) [139], the promotion of controlling consumed amounts (cf. [29]), and to better conduct healthier behaviors in practice (cf. [137]). However, research results show promising yet inconsistent outcomes regarding nurses’ physical activity and eating behaviors [78]. Nevertheless, in this context, behavioral interventions in terms of educating employees of outpatient care regarding back-friendly work is advised [140,141]. To decrease outpatient caregivers’ stress perceptions and the potentially increased risk of musculoskeletal disorders, interventions to strengthen their resilience could be aspirational, as they also promote individual personal resources [43]. Resilience interventions are already known from the care setting (e.g., [24,28,104,108,142,143]). This should continue to be strived for, and also in outpatient care, as employees’ stress perceptions and the risk of negative strain reactions are high [4], particularly in times of the still ongoing COVID-19 pandemic [144]. Since the experience of work stress favors presenteeism behavior, a higher degree of resilience could also contribute to a reduction in presenteeism, as this could assist with better dealing with potentially perceived stress (cf. [145]). Stress reduction has also been proven to have a reducing effect on presenteeism among healthcare workers [146]. Self-efficacy programmes can also be introduced in outpatient care, to target better individual health behavior patterns there, as has already been shown in other research findings (cf. [147,148]).

Employers from the outpatient care sector should offer interventions, such as resilience training and future programmes for health-promoting behaviors (e.g., smoking cessations, nutrition, reduction of stress perception) that are needs-based and that target specific groups of employees (cf. [17,149]). Finally, the professional monitoring of interventions can lead to increased effectiveness, and should therefore be pursued (cf. [150]). In order to ensure sufficient and appreciative communication in the outpatient care service between all parties involved, and thus to prevent possible perceptions of stress, regular communication training should be offered [151,152]. In this context, health-promoting leadership also becomes relevant, and should be given direct attention in WHP [153]. Accordingly, care managers could be trained by employers in such workshops.

Since the implementation of behavioral changes has been proven to be more difficult in practice, more attention should be paid to the structural level of WHP measures [69]. Recent research findings from inpatient care settings also show that participants found it difficult to focus on interventions with different goals at the same time [74]. Therefore, evaluations should be made on which intervention is most urgently needed for the individual staff member at the time under review, and this should be the focus for developing WHP measures in-house [154]. Furthermore, time seems to be an important influencing factor in the uptake of WHP interventions offered (cf. [47,48,111]). For this reason, employers should offer WHP measures not only for free, but they should also support their employees by offering interventions during working time (cf. [47,48]). For nurses, a mere 5 min a day has already been shown to have a significant reduction in stress perception [105]. Therefore, such interventions do not necessarily have to be scheduled for certain hours. At all times, however, employer support on a human and financial level is not to be neglected [47]. The provision of pedometers, moreover, can not only contribute to an increase in physical activity, but can also help with reducing stress perception. Using them would also not interfere with leisure time (cf. [32,80,83]). Moreover, employers need to encourage outpatient caregivers to take legally defined breaks (ArbZG [155]), which seemed to be quite difficult in the German care setting (cf. [42]). A sufficient break culture must be encouraged by care employers, to promote healthier behaviors (cf. [156,157]). In this regard, Wendsche [158] has recently published a checklist for examining the organization of breaks in care activities, which employers in outpatient care can use as an orientation. In addition, staff shortages should be tackled in the future, as they can also be a hindrance to WHP implementation [50]. Work-related demands in outpatient care (e.g., mobility requirements) as well as above-average break absences in the care sector [42] could indicate that a possible lack of spatial structures may make innovative digital offers of WHP in outpatient care appear sensible [19,110,129]. Irregular working hours, the unpredictability of filling in for colleagues when they are sick, and time pressure, are particular stress factors in outpatient care [127,132]. Digital measures of WHP can also be a supportive supplement in a work setting that is limited in time and place [17]. In connection with increasing digitalization, new forms of information and communication technologies appear to be more significant in the context of health promotion [159]. The low-threshold nature of access could also encourage the uptake of digital health promotion measures [160,161], as has already been positively highlighted by Bazarko, Cate, Azocar and Kreitzer [25]. Social networking via digital channels could also improve a possible lack of team spirit in outpatient care [162,163]. Specially developed mobile interventions, for example, in the form of apps, can effectively reduce the stress levels of employees and promote their well-being [164]. The stress-reducing effect of digital WHP interventions has already been underlined by Hersch, Cook, Deitz, Kaplan, Hughes, Friesen and Vezina [99] when examining the impact on stationary nursing staff. Moreover, further study results from the inpatient setting underline that target group-oriented eHealth interventions can support health-promoting behaviors [102]. In this context, the development of WHP interventions for use on smartphones, especially in the mobile outpatient setting, can be promising (cf. [17,165,166]).

A combination of health-promoting interventions at the behavioral and structural level should be pursued in the future, not only to strengthen personal resources and coping strategies, but also to optimize workplace conditions and adapt them to employees, which seems to be the most effective way [18,43,136,167], and is therefore considered to be desirable and promising [37,156]. For instance, with regard to back-friendly work, not only is education in theory needed, but the necessary tools have to be provided by the employer. In particular, employee groups need to be targeted (cf. [36]). According to a systematic review, a lack of WHP measures needs to be tackled, for instance, for older employees (cf. [168]), as the average age of outpatient caregivers in Germany is also constantly rising [13]. However, there are only a few studies in the scientific literature that address this issue of WHP for older workers (cf. [168]). Nonetheless, target group-specific WHP remains indispensable [17,154].

## 5. Conclusions

The present study is the first explorative and qualitative study for examining WHP measures in German outpatient care services, which is an as-yet unexplored field. Our respondents’ statements illuminated various WHP interventions that already exist, or that could be desirable with regard to their needs. Subjectively perceived barriers for implementing WHP measures illustrate the relevance of future changes in the work organization, and the creation of more healthy working conditions in outpatient care. WHP is not yet established in the care setting, as caregivers as a target group have not received enough attention so far. However, WHP seems promising for enhancing healthy behaviors and health status [169]. Based on our explorative interviews, we conclude that occupational health management must be more strongly established in the care setting to be able to guarantee the development, implementation, and evaluation of needs-based and targeted WHP.

## Figures and Tables

**Table 1:** Interview topic list.

Phase of the Interview	Contents
1. Information phase	Introduction: study information, confidentiality, informed consent
2 Warm-up phase	Qualifications, working activity
3. Main phase	Health impairment (e.g., “Do you perceive any impairment of your health due to your job?”) Workplace health promotion measures (e.g., “Are there any health promotion offers in your institution?”) Challenges in practice (e.g., “What are the challenges in implementing and using the offers”) Requests and needs (e.g., “Do you have any needs/wishes/suggestions for improving your work and health behaviour?”)
4. Final phase and end of the interview	Socio-demographics of the interviewees and farewell

**Table 2:** Participant characteristics.

		n
**Gender**		
	Female	21
	Male	9
**Age (years)**		
	20–30	6
	31–40	2
	41–50	10
	51–60	8
	≥61	4
**Qualification**		
	Health and medical nurse	3
	Physician assistant	2
	Geriatric nurse and paramedic	2
	Health and paediatric nurse	1
	Caregiver	10
	Geriatric nurse	9
	Home and family care	1
	Storekeeper	1
	Interior decorator	1
**Work experience (years)**		
	1–5	14
	6–10	6
	>10	10
**Work Schedule**		
	Full-time	21
	Part-time	9
**Nationality**		
	German	26
	Other	4

**Table 3:** Identified WHP among interviewed outpatient caregivers.

Identified WHP	Outpatient Caregivers (n)
**Offered WHP**	
Fruit basket	7
Smoking/alcohol cessation	8
Yoga	4
Sports courses	6
Company doctor	6
Financial contribution for the gym	5
PPE	2
**Further known WHP**	
Back college	7
Massage chair	4
Water dispenser	4
Nutrition counselling	2
Cooking with colleagues	1
**Use of digital offers**	
Pedometer	5
PMR	4

**Table 4:** Requests and needs of interviewed outpatient caregivers regarding further WHP measures.

Requests and Needs Regarding WHP Measures	Outpatient Caregivers (n)
Physical activity programme/subsidy for gym	12
Nutritional counselling	6
Provision of water in care service	4
Joint activities	6
Relaxation techniques (yoga, tai chi, massage)	7
Provision of work clothes	3

## Data Availability

The data analyzed during the current study are not publicly available due to German national data protection regulation. They are available on individual request from the corresponding author.
